# Activation of TLRs Triggers GLP-1 Secretion in Mice

**DOI:** 10.3390/ijms24065333

**Published:** 2023-03-10

**Authors:** Lorène J. Lebrun, Alois Dusuel, Marion Xolin, Naig Le Guern, Jacques Grober

**Affiliations:** 1 INSERM LNC UMR1231, Université de Bourgogne, 21000 Dijon, France; 2 LipSTIC LabEx, 21000 Dijon, France; 3 Institut Agro Dijon, 21000 Dijon, France

**Keywords:** glucagon-like peptide 1, toll-like receptors, polymicrobial infection, cecal ligation puncture, inflammation

## Abstract

The gastrointestinal tract constitutes a large interface with the inner body and is a crucial barrier against gut microbiota and other pathogens. As soon as this barrier is damaged, pathogen-associated molecular patterns (PAMPs) are recognized by immune system receptors, including toll-like receptors (TLRs). Glucagon-like peptide 1 (GLP-1) is an incretin that was originally involved in glucose metabolism and recently shown to be rapidly and strongly induced by luminal lipopolysaccharides (LPS) through TLR4 activation. In order to investigate whether the activation of TLRs other than TLR4 also increases GLP-1 secretion, we used a polymicrobial infection model through cecal ligation puncture (CLP) in wild-type and TLR4-deficient mice. TLR pathways were assessed by intraperitoneal injection of specific TLR agonists in mice. Our results show that CLP induces GLP-1 secretion both in wild-type and TLR4-deficient mice. CLP and TLR agonists increase gut and systemic inflammation. Thus, the activation of different TLRs increases GLP-1 secretion. This study highlights for the first time that, in addition to an increased inflammatory status, CLP and TLR agonists also strongly induce total GLP-1 secretion. Microbial-induced GLP-1 secretion is therefore not only a TLR4/LPS-cascade.

## 1. Introduction

There is an extensive interface between the gastrointestinal tract and its surrounding environment. Therefore, in addition to nutrient absorption, the gastrointestinal tract plays an important role as a barrier against the microbial components of the gut. Even if the gut microbiota is crucial for human physiology, with roles including non-digestible carbohydrate metabolism, antimicrobial protection and immunomodulation [1], it has to be kept separate from the rest of the inner body. Given these conditions, it is consistent that the gut hosts the most developed immune system in the organism: the gut-associated lymphoid tissues (GALTs) [2]. Alterations in the gut barrier and subsequent pathogen invasion activate inflammatory processes and can have disastrous consequences. When the gut barrier is damaged, the host’s innate immune system rapidly recognizes and responds to pathogen-associated molecular patterns (PAMPs) from microbes via specific receptors, including toll-like receptors (TLRs) [3], which induce immune cell recruitment and cytokine secretion.

Glucagon-like peptide 1 (GLP-1) is an incretin produced by enteroendocrine cells (EECs). It was originally described for its ability to stimulate insulin secretion and for its implication in glucose metabolism [4], but in addition to these glucoregulatory functions, GLP-1 also exerts anti-inflammatory effects [5]. GLP-1 agonists have been shown to have protective effects on the cardiovascular system [6,7,8], and, in humans, GLP-1 plasma levels are increased in sepsis and critical illness [9]. In patients undergoing cardiac surgery with cardiopulmonary bypass, a situation known to alter the gut barrier [10], we have shown that GLP-1 was associated with poor clinical outcomes [11].

In addition, it has been suggested that GLP-1 has a role in maintaining gut homeostasis [12], but the literature on this is scarce. Liraglutide, a GLP-1 agonist, has been shown to attenuate intestinal ischemia/reperfusion injury in mice [13]. Moreover, it was suggested to have a protective role in gut permeability [14]. GLP-1 secretion mechanisms have been extensively described [15], and gut microbiota is involved in these secretory pathways through different mechanisms. Short-chain fatty acids produced by gut bacteria increase GLP-1 secretion through G-protein-coupled receptors GPR41/GPR43 [16]. Microbe-metabolized secondary bile acids increase GLP-1 secretion [17]. Bacterial components such as peptides from *Akkermansia muciniphila* [18] and from *Staphylococcus epidermidis* [19] have also been associated with enhanced GLP-1 secretion. Moreover, we and others have previously shown that inflammatory bacterial-derived lipopolysaccharides (LPS) increase GLP-1 plasma levels [20,21]. LPS molecules are able to directly activate the TLR4 in enteroendocrine L cells, leading to a significant and rapid increase in GLP-1 secretion upon gut-barrier alterations [22]. This LPS/GLP-1 cascade underlines the critical role of EECs as the key mucosal sensor of gut injury [23].

In different studies aiming to mimic sepsis using the cecal ligation puncture (CLP), which is one of the most frequently used models, TLR expression was modulated in the lungs, liver, kidneys and intestines [24,25,26]. EECs express multiple TLRs [27] and are targets of pro-inflammatory cytokines such as interleukin-6 (IL6) and tumor necrosis factor alpha (TNFA), which are known to stimulate GLP-1 secretion [15]. The aim of this study was therefore to investigate whether, besides TLR4, the only TLR involved to date in GLP-1 secretion, other TLRs could also be implicated in the control of GLP-1 secretion. For this purpose, the CLP model was performed in wild-type (WT) and TLR4-deficient (*Tlr4*^-/-^) mice. In addition, after mice were injected with TLR-specific agonists, we assessed cytokines, TLR expression and GLP-1 secretion. Our data suggest for the first time that the activation of TLRs other than TLR4 is involved in GLP-1 secretion.

## 2. Results

### 2.1. CLP Induces Systemic Inflammation and Modulates Inflammatory Gene Expression in the Gut

**Figure 1:**
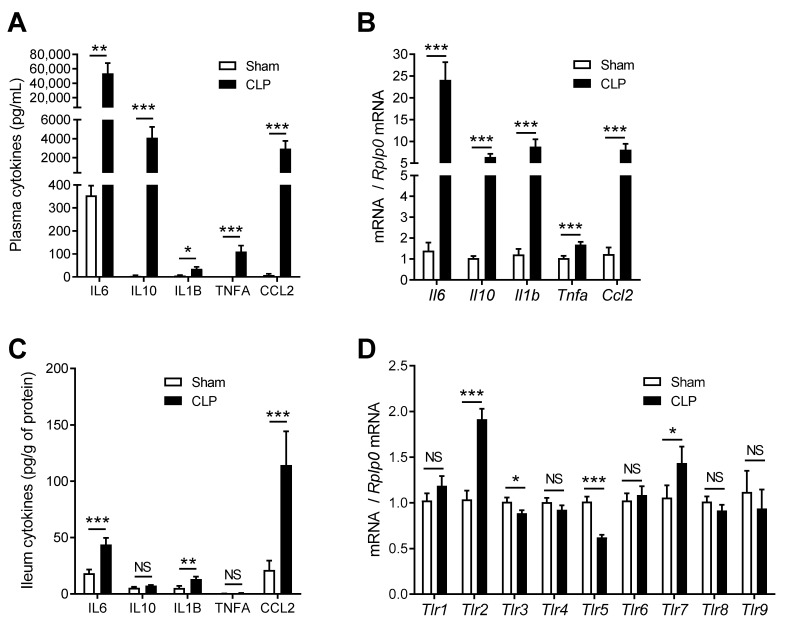
Cecal ligation puncture (CLP) induces systemic inflammation and modulates inflammatory gene expression in the gut. Evaluation of the inflammatory status of mice 3 h after the onset of CLP or in sham control mice (*n* = 10). (**A**) Levels of plasma cytokines (pg/mL): interleukin-6 (IL6), interleukin-10 (IL10), interleukin-1 beta (IL1B), tumor necrosis factor alpha (TNFA) and C-C motif chemokine ligand 2 (CCL2). (**B**) Quantification of cytokine mRNA levels (compared to ribosomal protein lateral stalk subunit P0 (*Rplp0*) mRNA) in the ileum. (**C**). Levels of ileum cytokines (pg/g of protein). (**D**). Quantification of toll-like receptor (TLR) mRNA levels (compare to *Rplp0* mRNA) in the ileum. Statistical analyses were performed using the matched Student’s *t*-test or Mann–Whitney test. NS, not significant; * *p* < 0.05, ** *p* < 0.01 and *** *p* < 0.001. All results are expressed as mean ± SEM.

The quantification of plasma cytokines revealed a major rise in inflammatory markers after CLP (Figure 1A). Cytokine mRNA levels were significantly increased in the ileum of CLP-treated mice compared to sham mice (Figure 1B). An analysis of the cytokines in the ileum showed that IL6, interleukin-1 beta (IL1B) and C-C motif chemokine ligand 2 (CCL2) were significantly increased after CLP (Figure 1C). The mRNA levels in TLRs (1 to 9) were then assessed in the gut after CLP treatment (Figure 1D), showing a significant induction of *Tlr2* and *Tlr7* mRNA levels.

### 2.2. TLR Agonists Increase Cytokines and Expression of TLRs

**Figure 2:**
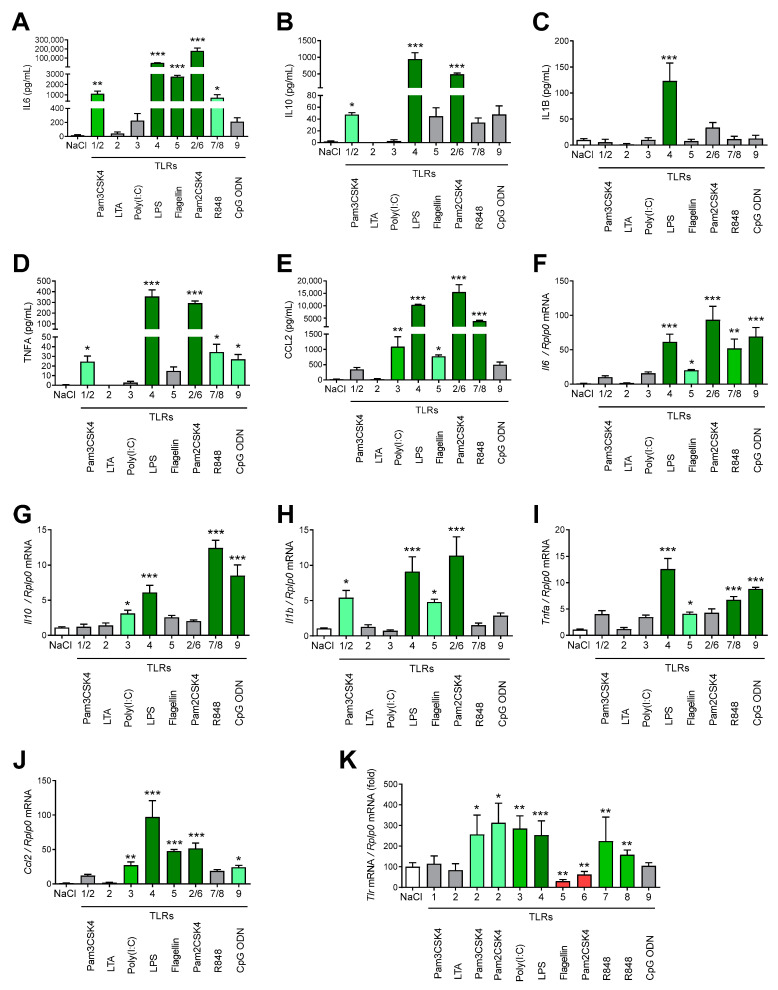
Toll-like receptor (TLR) agonists increase cytokines and *Tlr* expressions. Evaluation of the inflammatory status in mice 3 h after intraperitoneal injection of sodium chloride (NaCl) (control group; *n* = 9) or TLR agonists: Pam3CysSerLys4 (Pam3CSK4; TLR1/2 agonist; 1 mg/kg; *n* = 5), lipoteichoic acid (LTA; TLR2 agonist; 1 mg/kg; *n* = 5), Pam2CysSerLys4 (Pam2CSK4; TLR2/6 agonist; 1 mg/kg; *n* = 5), polyinosinic–polycytidylic acid (Poly(I:C); TLR3 agonist; 1 mg/kg; *n* = 5), lipopolysaccharides (LPS; TLR4 agonist; 1 mg/kg; *n* = 9), flagellin (TLR5 agonist, 1 mg/kg; *n* = 5), resiquimod (R848; TLR7/8 agonist; 1 mg/kg; *n* = 5) and CpG oligodeoxynucleotides (CpG ODN; TLR9 agonist; 0.6 mg/kg; *n* = 5). (**A**–**E**) Levels of plasma cytokines (pg/mL): interleukin-6 (IL6), interleukin-10 (IL10), interleukin-1 beta (IL1B), tumor necrosis factor alpha (TNFA) and C-C motif chemokine ligand 2 (CCL2). (**F**–**J**) Quantification of cytokines mRNA levels in the ileum (compared to ribosomal protein lateral stalk subunit P0 (*Rplp0*) mRNA): *Il6, Il10, Il1b, Tnfa* and *Ccl2*. (**K**) Quantification of *Tlr* mRNA levels in the ileum (compared to *Rplp0* mRNA); results are expressed as fold induction of the NaCl group. Statistical analyses were performed using the Kruskal–Wallis test; * *p* < 0.05, ** *p* < 0.01 and *** *p* < 0.001. All results are expressed as mean ± SEM.

Intraperitoneal injections of different TLR agonists induced inflammatory status and increased TLR gene expression in the gut (Figure 2). Plasma levels of pro- or anti-inflammatory cytokines were assessed (Figure 2A–E). Most of the analyzed cytokines were increased upon stimulation with specific TLR agonists. One can note that only TLR4, through its activation by LPS, led to an increase in all the cytokines. The mRNA gene expression of these cytokines was then measured in the ileum (Figure 2F–J). Here again, the mRNA expression of many cytokines increased through activation by TLRs, with TLR4 being the receptor that induced increases in all mRNA levels. Lastly, mRNA gene expression of the different TLRs was quantified in the ileum (Figure 2K). Among the nine studied TLRs, four had either a reduced (*Tlr5* and *Tlr6*) or an identical (*Tlr1* and *Tlr9*) mRNA expression.

### 2.3. GLP-1 Secretion Is Mediated through Multiple TLRs

**Figure 3:**
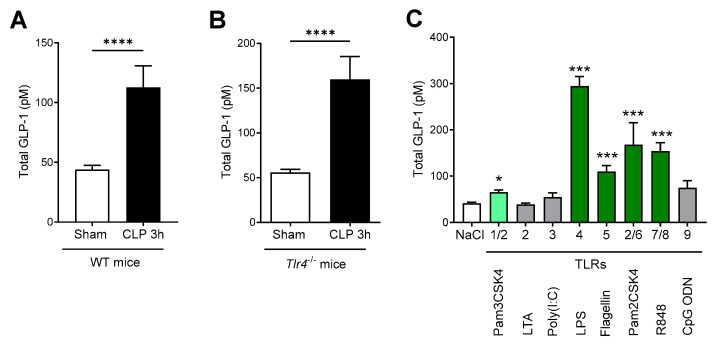
Glucagon-like peptide 1 (GLP-1) secretion is mediated through multiple toll-like receptors (TLRs). (**A**) Total GLP-1 plasma levels (pM) 3 h after the onset of cecal ligation puncture (CLP; *n* = 10) or in sham control mice (*n* = 10) in wild-type (WT) mice. (**B**) Total GLP-1 plasma levels (pM) 3 h after the onset of CLP (*n* = 11) or in sham control mice (*n* = 10) in TLR4-deficient (*Tlr4*^-/-^) mice. (**C**) Total GLP-1 plasma levels in mice 3 h after an intraperitoneal injection of sodium chloride (NaCl; control group; *n* = 9) or different TLR agonists: Pam3CysSerLys4 (Pam3CSK4; TLR1/2 agonist; 1 mg/kg; *n* = 5), lipoteichoic acid (LTA; TLR2 agonist; 1 mg/kg; *n* = 5), Pam2CysSerLys4 (Pam2CSK4; TLR2/6 agonist; 1 mg/kg; *n* = 5), polyinosinic-polycytidylic acid (Poly(I:C); TLR3 agonist; 1 mg/kg; *n* = 5), lipopolysaccharides (LPS; TLR4 agonist; 1 mg/kg; *n* = 9), flagellin (TLR5 agonist, 1 mg/kg; *n* = 5), resiquimod (R848; TLR7/8 agonist; 1 mg/kg; *n* = 5) and CpG oligodeoxynucleotides (CpG ODN; TLR9 agonist; 0.6 mg/kg; *n* = 5). Statistical analyses were performed using the Mann–Whitney test (CLP experiment) or the Kruskal–Wallis test (agonists experiment); * *p* < 0.05, *** *p* < 0.001 and **** *p* < 0.0001. All results are expressed as mean ± SEM.

Total plasma GLP-1 concentrations were assessed in CLP-induced and sham control mice. As shown in Figure 3A, CLP significantly increased GLP-1 secretion 3 h after the onset of CLP. The increased secretion was maintained at 6 and 24 h (data not shown). In *Tlr4^-/-^* mice, meaning in the absence of a functional TLR4 receptor, CLP still induced a significant increase in GLP-1 secretion compared to sham control mice (Figure 3B), suggesting the involvement of TLRs other than TLR4 in CLP-induced GLP-1 secretion. The total GLP-1 plasma levels were also investigated after the injection of different TLRs agonists compared to sodium chloride (NaCl)-injected mice (Figure 3C). Most of the TLRs agonists tested were able to induce the secretion of GLP-1.

## 3. Discussion

Here, we show that a CLP model induces a rapid (3 h) and significant increase in GLP-1 plasma levels and that this effect is associated with changes in TLR gene expression in the ileum. Moreover, CLP-induced GLP-1 secretion also occurs in *Tlr4^-/-^* mice, suggesting that PAMPs other than LPS induce a significant increase in GLP-1 secretion through the activation of TLRs.

GLP-1 is a gut-derived hormone generally described in the literature as playing an important role in glucose homeostasis by increasing insulin secretion, suppressing glucagon expression and also inhibiting gastric emptying and reducing food intake. GLP-1 is produced by EECs, which represent only 1% of intestinal epithelial cells. Despite this small proportion, the hormonal secretion of EECs is essential for the coordination of food intake, digestion and absorption [28]. However, an intestinal immuno-endocrine axis has been described for EECs, which may play a key role in the gut immune response to both pathogens and commensal bacteria [23]. Moreover, inflammatory bowel diseases (IBDs) are associated with an increase in the number of these cells in the ileum [29]. Cells from the gut’s immune system express a wide range of peptide receptors produced by EECs [30]: ghrelin increases the activation and proliferation of T lymphocytes [31], and cholecystokinin stimulates the production of acetylcholine by B cells and thus has an effect on neutrophil recruitment [32]. In addition, both in vitro and in vivo studies demonstrate that EECs also express certain TLRs [27,33], and the activation of these TLRs induce hormone secretions: GLP-1 after TLR4 activation [22]; and cholecystokinin after TLR4, TLR5 and TLR9 activation.

GLP-1 has also been described as an endogenous immune modulator with a wide range of physiological effects, such as stimulation of anti-inflammatory signaling [5]. In two previous studies, we showed that (i) pro-inflammatory molecules located on the surface of Gram-negative bacteria, LPS, are able to enhance GLP-1 plasma levels when injected into mice or humans; and (ii) when the gut barrier is damaged after a gut ischemia/reperfusion experiment, endogenous LPS naturally present in the gut are involved in this rapid GLP-1 secretion, at least partly through the activation of TLR4 receptors [20,22]. Why GLP-1 is released upon gut-barrier dysfunction is currently unknown. Contrary to GLP-2, whose role in gut homeostasis and, in particular, gut permeability is well documented [34], the literature regarding GLP-1 in intestinal homeostasis and, in particular, in regulating the barrier function is scarce [35]. Different pathological situations known to cause a gut-barrier disturbance are associated with enhanced GLP-1 secretion. For instance, in IBDs such as Crohn’s disease or ulcerative colitis, GLP-1 plasma levels are dysregulated [36]. In IBD patients, GLP-1 serum levels are increased [37]. Compared to healthy controls, Crohn’s disease patients have upregulated fasting GLP-1 levels, while stimulated GLP-1 secretion is reduced [38]. Sepsis is another situation in which GLP-1 secretion is enhanced. Previous studies have demonstrated that the endogenous GLP-1 system is activated during sepsis [9] and have associated GLP-1 levels with predicted mortality [39,40].

The CLP model used in this study is a widely used animal model for sepsis [41]. Gut-derived sepsis causes intestinal barrier dysfunction and induces bacterial translocation and the development of multiple-organ-dysfunction syndrome [42]. Whether in this study or in our previous one [22], the results obtained with *Tlr4*^-/-^ mice suggest that other molecules derived from the gut, and certainly other TLRs, are involved in the GLP-1 secretion that occurs rapidly after gut-barrier alterations. Previous studies have suggested an important role for TLRs in the regulation of gut permeability during infection [43]. Nevertheless, not all TLRs have the same effect on gut permeability. Whereas TLR1 and TLR2 signaling sustain epithelial integrity [44,45], the activation of epithelial TLR4 increases barrier permeability and leaky gut in both human cell lines and mouse models [46,47,48]. However, the presence of functional TLR4 receptors has been shown to protect the intestinal mucosa from damage induced by ischemia/reperfusion treatment in mice [49,50]. Alterations in the intestinal epithelium of mice treated with dextran sodium sulphate (DSS) are associated with a strong increase in intestinal permeability [51], and the subsequent repair capacities of the intestinal mucosa are reduced by the antagonization of the TLR4 receptor. *Tlr4*^-/-^ mice are also more sensitive to DSS and show greater bacterial translocation than WT mice [52]. Overall, these studies suggest that LPS/TLR4 engagement may provide beneficial effects in terms of gut-barrier integrity. Interestingly, in the experimental conditions described here, we observed the most robust GLP-1 secretion with LPS, the TLR4 agonist.

As discussed above, GLP-1 exerts anti-inflammatory properties and could contribute to gut-barrier regulation. Mice with a GLP-1 receptor deficiency are more sensitive to DSS-induced intestinal injuries [12]. One may assume that LPS-induced GLP-1 secretion could contribute to the beneficial effects of TLR4 and, more largely, PAMP-induced GLP-1 secretion. Moreover, TLR2-, TLR5- and TLR9-deficient mice are also more susceptible to chemically induced colitis [45,53,54]. Considering the anti-inflammatory properties of GLP-1, treatments for several disorders are based on the administration of GLP-1 agonists. For instance, in type 2 diabetes patients, semaglutide and liraglutide are associated with a reduction in cardiovascular events [6,55]. In preclinical studies of IBD, GLP-1 agonists have been shown to improve colitis by significantly improving the colon’s weight-to-length ratio, lowering the histopathological score and reducing pro-inflammatory cytokines levels [56,57]. A recent study has shown that chronic pretreatment with liraglutide in mice challenged with intestinal ischemia increased their overall survival [13]. In a human case report, the treatment of IBD (ulcerative colitis) patient with liraglutide led to a full remission of colitis symptoms [58]. In type 2 diabetes patients treated with GLP-1-based therapies, the disease course of IBD is improved compared with patients taking other antidiabetics [59]. Liraglutide treatment of mice undergoing sepsis induction improved the cardiovascular consequences of the dysregulated inflammation observed in sepsis [60]. Sepsis is associated with a hyperglycemic state that needs to be tightly controlled [61]. Indeed, hyperglycemia increases pathogen invasion [62] and, thus, nosocomial mortality [63]. GLP-1 agonists are currently under investigation as a means of controlling hyperglycemia and inflammation during sepsis [64]. Besides the exogenous administration of GLP-1 agonists, the increase of endogenous GLP-1 secretion can also be considered from a therapeutic point of view. For instance, probiotics are known to promote GLP-1 secretion and alleviate both type 2 [65,66] and type 1 [67] diabetes symptoms. Probiotic-related increases in GLP-1 secretion and the associated beneficial effects can be mediated through short-chain fatty acid production [68].

The present study has some limitations. It focuses mainly on TLR activation, and we cannot exclude that other pathways/receptors are involved. Indeed, muramyl dipeptide, a bacterial component, has been shown to improve insulin sensitivity and glucose tolerance in diet-induced obese mice via an increase in GLP-1 secretion by acting through the nucleotide oligomerization domain 2 (NOD2) receptor [69]. Moreover, it is well established (and observed in the present study) that pro-inflammatory cytokines are secreted upon activation of TLRs. Since it has been demonstrated that IL6 and TNFA induce the secretion of GLP-1 [15], we can assume that the observed TLR-induced GLP-1 secretion could be indirectly mediated through cytokine expression. In this study, both the CLP model and the injection of TLR agonists induced cytokine release. Therefore, whether the above-described GLP-1 secretion is a direct result of TLR activation of EECs or an indirect pathway still needs to be determined. It is generally assumed in the literature that TLR ligands are able to induce GLP-1 secretion even though, to date, only the LPS/TLR4 pathway has been demonstrated to be involved in this secretion [20,22]. This study is the first to clearly highlight that the activation of other TLRs also leads to a GLP-1 secretion.

## 4. Materials and Methods

### 4.1. Experimental Animals and Samplings

Male wild-type (WT) C57BL/6J mice and male mice deficient in TLR4 (*Tlr4*^-/-^) on C57BL/6J background were used. Mice were 8-to-10 weeks of age and were housed in a controlled environment (light from 7 am to 7 pm, constant humidity and temperature) and were given a standard chow diet (A03 diet, Safe SAS, Augy, France). Every sampling procedure was performed under inhaled anesthesia (isoflurane) titrated to maintain spontaneous breathing. Blood was collected by intracardiac puncture in EDTA-coated tubes. Plasmas were separated by centrifugation at 8000× *g* for 10 min at 4 °C and were stored at −20 °C until use. Ileum tissues were immediately snap-frozen (immersion in liquid nitrogen) after harvest and stored at −80 °C until utilization.

### 4.2. Cecal Ligation Puncture (CLP) Treatment

CLP treatment was performed as previously described [70] on mice under inhaled anesthesia (isoflurane) titrated to maintain spontaneous breathing. Anesthetized mice were placed on a heating pad during the surgical procedure. Mice abdomens were shaved and cleaned with alcohol. A midline laparotomy was made, the cecum was isolated and ligated below the ileocecal valve with a 4–0 suture and without causing bowel obstruction. A perforation was made in the ligated cecum by a single puncture with a 21-gauge needle and gently squeezed to externalize a small amount of fecal matter. Cecum was then returned into the peritoneal cavity. The abdominal cavity of the mice was closed by suture and wound clips. Mice were then resuscitated with a subcutaneous injection of 0.4 mL of NaCl.

### 4.3. Drugs Administration in Mice

Mice were fasted for 4 h and intraperitoneally injected with different TLRs agonists (10µL/g): Pam3CysSerLys4 (Pam3CSK4; TLR1/2 agonist; 1 mg/kg; tlrl-pms, Invivogen, San Diego, CA, USA), lipoteichoic acid (LTA; TLR2 agonist; 1 mg/kg; tlrl-pslta, Invivogen, San Diego, CA, USA), Pam2CysSerLys4 (Pam2CSK4; TLR2/6 agonist; 1 mg/kg; tlrl-pm2s, Invivogen, San Diego, CA, USA), polyinosinic-polycytidylic acid (Poly (I:C); TLR3 agonist; 1 mg/kg, tlrl-picw, Invivogen, San Diego, CA, USA), lipopolysaccharides (LPS; TLR4 agonist; 1 mg/kg; tlrl-pb5lps, Invivogen, San Diego, CA, USA), flagellin (TLR5 agonist, 1 mg/kg; tlrl-bsfla, Invivogen, San Diego, CA, USA), resiquimod (R848; TLR7/8 agonist; 1 mg/kg; tlrl-r848, Invivogen, San Diego, CA, USA) and CpG oligodeoxynucleotides (CpG ODN; TLR9 agonist; 0.6 mg/kg; tlrl-dls01, Invivogen, San Diego, CA, USA).

### 4.4. Real-Time Quantitative PCR

Ileum was immediately snap-frozen (immersion in liquid nitrogen) after harvest and stored at −80 °C until RNA extraction. Total RNA was isolated using an RNA extraction kit (RNeasy Mini Kit, 74106, Qiagen, Hilden, Germany). RNA extraction included a DNAse treatment step. RNA was quantified using the NanoDrop 1000 spectrophotometer (ThermoFisher Scientific, Waltham, MA, USA), and 500 ng of RNA from each sample was reverse transcribed using the High-Capacity cDNA Reverse Transcription Kit (Multiscribe^®^ reverse transcriptase, 4368813, Applied Biosystems, Waltham, MA, USA) according to the manufacturer’s instructions. Quantitative PCRs were performed using StepOnePlus (Real-Time PCR System, Applied Biosystems, Waltham, MA, USA) and TaqMan^®^ technology (4324018, Applied Biosystems, Waltham, MA, USA). The mRNA level was normalized to levels of *Rplp0* mRNA, and the results were expressed as relative expression levels, using the 2-ΔΔCt method.

### 4.5. Plasma and Tissues Biochemical Analyses

Cytokines and total GLP-1 concentrations were determined by commercially available ELISA Kits (M-CYTOMAG-70K, Millipore, Burlington, MA, USA; EZGLP1T-36K, Millipore, Burlington, MA, USA). Ileum segments were homogenized in RIPA buffer (89901, ThermoFisher Scientific, Waltham, MA, USA) containing antiproteases and antiphosphatases (11873580001, Roche, Penzberg, Germany). Total protein concentration was measured with a commercial colorimetric assay kit (Thermo Scientific™ Pierce™ BCA assay kit, Waltham, MA, USA).

### 4.6. Statistics

Data were collected using Microsoft Excel for Office 365. Data are presented as means ± SEM. Statistical analyses were performed using Prism 6.0 (GraphPad, San Diego, CA, USA). To decide whether to use parametric or non-parametric statistics, the normality of distributions were assessed with the Shapiro–Wilk test (under *n* = 7, distributions were considered to be non-normal). The statistical significance of differences between two groups was evaluated with the Mann–Whitney U test or Student’s *t*-test (a statistical correction was applied when variances were different between groups). For more than two groups, a Kruskal–Wallis test was performed. A value of *p* < 0.05 was considered statistically significant (NS, not significant; * *p* < 0.05, ** *p* < 0.01, *** *p* < 0.001 and **** *p* < 0.0001).

## 5. Conclusions

Overall, we report that activation of TLRs either through a polymicrobial infection model (CLP) or with specific agonists leads to increases in GLP-1 secretion. We confirm the secretory effects of LPS, and we extend this secretory capacity to others PAMPs through the activation of other TLRs than TLR4. The multiple-TLRs-induced GLP-1 secretion observed in this study supports a tiny link between GLP-1 and gut inflammatory processes. Even if the secretion of GLP-1 has increased the association to inflammatory processes such as IBD or sepsis, its actual systemic and gut functions in this context need more investigations.

## Data Availability

The authors confirm that the data supporting the findings of this study are available within the article.
